# ^1^H-NMR, ^1^H-NMR T_2_-edited, and 2D-NMR in bipolar disorder metabolic profiling

**DOI:** 10.1186/s40345-017-0088-2

**Published:** 2017-06-04

**Authors:** Sumit Sethi, Mariana Pedrini, Lucas B. Rizzo, Maiara Zeni-Graiff, Caroline Dal Mas, Ana Cláudia Cassinelli, Mariane N. Noto, Elson Asevedo, Quirino Cordeiro, João G. M. Pontes, Antonio J. M. Brasil, Acioly Lacerda, Mirian A. F. Hayashi, Ronei Poppi, Ljubica Tasic, Elisa Brietzke

**Affiliations:** 1 Department of Psychiatry, Universidade Federal de São Paulo-UNIFESP, Rua Borges Lagoa, 570. Vila Clementino, São Paulo, CEP 04038-020 Brazil; 2 Department of Pharmacology, Universidade Federal de São Paulo-UNIFESP, Rua Três de Maio, 100. Vila Clementino, São Paulo, CEP 04044-020 Brazil; 3 Department of Psychiatry, Irmandade da Santa Casa de Misericórdia de São Paulo (ISCMSP), Rua Major Maragliano, 287. Vila Mariana, São Paulo, CEP 04017-030 Brazil; 4 Laboratório de Química Biológica, Department of Organic Chemistry, Institute of Chemistry, Universidade Estadual de Campinas-UNICAMP, Caixa Postal 6154, Campinas, São Paulo, CEP 13083-970 Brazil; 5 Department of Analytical Chemistry, Institute of Chemistry, Universidade Estadual de Campinas-UNICAMP, Caixa Postal 6154, Campinas, São Paulo, CEP 13083-970 Brazil

**Keywords:** ^1^H-NMR, Biomarkers, Bipolar disorder, Metabolic profiling, Chemometrics, 2D NMR

## Abstract

**Background:**

The objective of this study was to identify molecular alterations in the human blood serum related to bipolar disorder, using nuclear magnetic resonance (NMR) spectroscopy and chemometrics.

**Methods:**

Metabolomic profiling, employing ^1^H-NMR, ^1^H-NMR T_2_-edited, and 2D-NMR spectroscopy and chemometrics of human blood serum samples from patients with bipolar disorder (*n* = 26) compared with healthy volunteers (*n* = 50) was performed.

**Results:**

The investigated groups presented distinct metabolic profiles, in which the main differential metabolites found in the serum sample of bipolar disorder patients compared with those from controls were lipids, lipid metabolism-related molecules (choline, *myo*-inositol), and some amino acids (*N*-acetyl-l-phenyl alanine, *N*-acetyl-l-aspartyl-l-glutamic acid, l-glutamine). In addition, amygdalin, α-ketoglutaric acid, and lipoamide, among other compounds, were also present or were significantly altered in the serum of bipolar disorder patients. The data presented herein suggest that some of these metabolites differentially distributed between the groups studied may be directly related to the bipolar disorder pathophysiology.

**Conclusions:**

The strategy employed here showed significant potential for exploring pathophysiological features and molecular pathways involved in bipolar disorder. Thus, our findings may contribute to pave the way for future studies aiming at identifying important potential biomarkers for bipolar disorder diagnosis or progression follow-up.

**Electronic supplementary material:**

The online version of this article (doi:10.1186/s40345-017-0088-2) contains supplementary material, which is available to authorized users.

## Background

Bipolar disorder (BD) is a potentially debilitating mental disorder affecting 1–3% of the population worldwide (Goodwin and Jamison 2007; Marohn 2011; Nierenberg et al. 2013; Phillips and Kupfer 2013). Previous studies have suggested a number of theories to explain the etiology of BD, including the influence of genetic factors, deficits in neurodevelopmental processes, implication of neurodegenerative pathologies, changes in monoamine neurotransmission, and abnormalities in neuroplasticity (van Enkhuizen et al. 2015; Goes 2016; Passos et al. 2016). A number of medications have been used for several decades to treat this disorder and/or to ameliorate the symptoms, though the mechanisms of action of most of mood stabilizers still remain to be fully elucidated (Malhi et al. 2013; Yatham et al. 2013). Increased accuracy in early diagnosis of BD is the key to improve the mental health outcomes, to ameliorate the clinical course, and to make better the treatment response of patients with this disorder. However, the diagnosis of BD, especially in early stages and in predominantly depressive presentations, remains challenging (Brietzke et al. 2016). These challenges could possibly be overcome by the identification of differentially expressed biomarkers, which could reflect or convey the pathophysiological processes (Oswald et al. 2007; Marmol 2008).

Hydrogen-1 nuclear magnetic resonance (^1^H-NMR) spectroscopy-based metabolomics can be used to monitor a wide range of metabolites in biological samples, allowing for a sensitive, high-throughput molecular screening (Beckonert et al. 2007). ^1^H-NMR has an exceptional reproducibility and is quantitative to the extent that a given peak area is directly proportional to the concentration of the corresponding metabolite, turning this technique a well-established approach for metabolomics (Maher et al. 2008). Although many platforms for metabolic profiling (for instance, the LC–MS, GC–MS, NMR, capillary electrophoresis-MS) are available, none technology alone provides the complete coverage of the metabolome. Previous employment of these techniques to study the brain of patients with psychiatric disorders was reported (Lan et al. 2009; Strzelecki et al. 2015), but it was scarcely applied for serum metabolome studies of individuals with mental illnesses.

In this study, a NMR metabolomics profiling approach was used to identify molecular alterations in the human blood serum of BD patients. Our intention was to conduct analysis to distinguish the individuals of type 1 BD patients and healthy control groups, based on their metabolic profiles. Also, as previously reported by us (Sussulini et al. 2009), with time distance and new sampling, NMR-based metabolomics has been applied as to verify if it is possible to be used for precise classification of aforementioned illness.

## Methods

### Studied population

Twenty-six euthymic outpatients with bipolar disorder (BD) type 1 (BD group) and 50 healthy control (HC) individuals (control group) were included in the research. All individuals in the BD group, with age between 18 and 65 years old, fulfilled the DSM-IV (Diagnostic and Statistical Manual of Mental Disorders) criteria according to SCID-I (Structured Clinical Interview for DSM Disorder). Euthymia was defined as not fulfill criteria for any mood episode and present, at the same time, with scores below 8 in Young Mania Rating Scale (YMRS) and Hamilton Depression Rating Scale (HDRS)-17 items. Exclusion criteria were as follows: be acutely suicidal, comorbidity with substance use disorders, except nicotine, presence of acute or unstable or chronic general medical comorbidity, inability to read and understand study procedures, pregnancy, and postpartum period. All subjects were inquired on medical history, including lifetime use of any medication. Body mass index (BMI) was also measured using the formula BMI = Weight (kg)/Height (m)^2^.

For comparison, a control group of 50 healthy volunteers was recruited from the community. To be eligible for the HC group, individuals were required to have age between 18 and 65 years old, negative history of any psychiatric condition current or along lifetime, and never made use of psychiatric medication. In addition, only volunteers with no family history of major mental disorders (schizophrenia-spectrum disorders, mood disorders, and suicide) were included. The same exclusion criteria of BD group were applied to HC group.

Clinical assessment included SCID-I for confirmation of diagnosis and assessment of psychiatric comorbidities. YMRS and HDRS-17 items were used to determine severity of manic and depressive symptoms, respectively. Functioning was estimated by General Assessment of Functioning (GAF).

### Serum collection and storage

All blood samples were taken in the morning (between 8 and 10 a.m.) after 12 h of fasting. Blood was drawn into Vacutainer tubes, and immediately allowed to clot for at least 30 min at room temperature, before it was centrifuged at 1500×*g*, for 5 min. The serum was then aliquoted, transferred into clean polypropylene tubes, and stored at −80 °C until use. The maximum period of storage was of two weeks.

### NMR spectroscopy analyses: ^1^H-NMR, ^1^H-NMR T_2_-edited, and 2D NMR

For NMR spectroscopy analyses, serum samples were thawed and centrifuged at 12,300×*g*, for 10 min, at 4 °C to separate any precipitate. Aliquots of 250 μL of the supernatants were diluted with 250 μL of deuterated water (D_2_O) or PBS buffer (250 μL containing 10% D_2_O) and transferred into 5.0 mm diameter NMR tubes. NMR tubes were placed into a Bruker 600 NMR spectrometer (Bruker Advance III, Bruker GmBH, Rheinestennen, Germany) using TBI (Triple resonance Broadband Inverse) probe at 25 °C. ^1^H-NMR spectra were recorded as three independent measurements for each sample using the CH_3_-lactate signal (δ1.33, 3H, d, ^*3*^ *J* = 7.0 Hz) as a reference, and applying the pulse sequence WATERGATE (p3919gp) with 128 ns (Piotto et al. 1992). T_2_-edited NMR spectra were recorded using the CPMG (Carr-Purcell-Meiboom-Gill) sequence (Meiboom and Gill 1958), where a fixed total spin–spin relaxation delay 2*nτ* of 100 ms was used to attenuate the broad NMR signals from slowly tumbling molecules such as proteins and lipids retaining those from low molecular weight compounds and some lipid components. For each spectrum, 128 transients were acquired into 32,000 data points, with a spectral width of 12 kHz. To confirm the assignments made from ^1^H-NMR and T_2_-edited spectra, some blood serum samples were also examined using 2D [^1^H–^13^C] HSQC. For each 2D spectrum, 256 increments with 64 transients per increment were collected and extended to 4 K data points. The signal assignments were based on the literature and/or databases such as Biological Magnetic Data Bank (BMRB) (Ulrich et al. 2008) and Human Metabolome Database (HMDB) (Wishart et al. 2007) and are indicated on the T_2_-edited spectrum and confirmed by the 2D fully assigned [^1^H–^13^C] HSQC data.

### Chemometrics of ^1^H NMR spectral data

^1^H-NMR data were transported to a data matrix, and chemometrics analysis, based on principal component analysis (PCA) and partial least-squares discriminant analysis (PLS-DA), were performed using MATLAB (The MathWorks, Natick, MA) or Pirouette (Infometrix, USA) software. T_2_-edited spectra were not used in chemometric analysis. The spectral region of chemical shifts, from 1.00 to 4.40 ppm, was used in chemometrics (Nørgaard 2009). PLS-DA was used as a supervision method for classification (Sena et al. 2004). All variables were auto scaled.

## Results

The summary of the collected sample characteristics is presented in Table 1. A predominance of females over males was observed in our BD sample, and the percentages were statistically different between the BD and HC groups. In addition, we observed, as expected, a high prevalence of tobacco smoking individuals in our BD group compared with HC group. Functioning was also impaired in the BD group compared with HC group, which reflects a well-known incomplete association between mood symptoms and functionality in individuals with BD. In addition, there was a clear predominance of chronic patients, with a mean duration of illness of about 15 years.

**Table 1:** Clinical and demographic characteristics of the sample

	Healthy controls (*n* = 50)	BD (*n* = 26)	Test	
			U or *X* ^2^	*p* value
Age in years; mean (SD)	35.69 (13.03)	40.38 (12.66)	187.000	0.261
Sex female; *N*(%)	17 (34)	17 (65)	5.114	0.023
Ethnicitycaucasian; *N*(%)	18 (36)	14 (53)	0.391	0.531
Years of education; mean (SD)	12.1 (3.8)	12.4 (3.6)	181.500	0.865
BMI in kg/m^2^; mean (SD)	26.2 (4.43)	28.06 (7.58)	170.500	0.507
Hours of physical exercise per week; mean (SD)	1.88 (2.44)	2 (2.93)	176.500	0.949
Current smoking; *N*(%)	3 (11.5)	4 (23.5)	1.084	0.297
Young mania rating scale; mean (SD)	0.5 (1.06)	0.7 (1.35)	208.500	0.661
Hamilton depression rating scale; mean (SD)	1.53 (1.96)	3.23 (3.84)	167.500	0.167
General functioning assessment (symptoms); mean (SD)	88.84 (6.97)	80 (10.15)	109.500	0.004
General functioning assessment (functioning); mean (SD)	87.69 (4.52)	75.88 (16.12)	127.000	0.011
Age at onset in years; mean (SD)	–	24.83 (9.83)	–	–
Duration of illness in years; mean (SD)	–	15.44 (12.69)	–	–
Number of total mood episodes; mean (SD)	–	9.05 (9.52)	–	–
Number of past manic episodes; mean (SD)	–	3.38 (3.82)	–	–
Number of past hypomanic episodes; mean (SD)	–	1.61 (4.11)	–	–
Number of past depressive episodes; mean (SD)	–	4.05 (5.47)	–	–
Number of past mixed episodes; mean (SD)	–	0 (0)	–	–
Age of diagnosis; mean (SD)	–	31.16 (11.11)	–	–
Age of first use of psychiatric medication; mean (SD)	–	26.55 (10.03)	–	–
Age of first use of mood stabilizer; mean (SD)	–	31.58 (11.78)	–	–

## ^1^H NMR spectroscopy data analysis

**Fig. 1:**
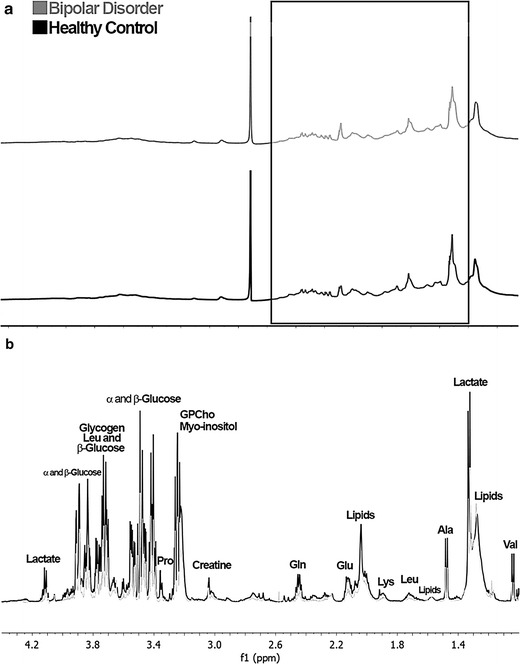
**a** ^1^H NMR Spectra with water suppression (Pulse sequence: p3919gp, ns = 128) blood serum sample from HC (*black*), and BD group (*gray*). Spectral region highlighted δ 4.40 to 1.00 with higher loadings. **b** ^1^H NMR Spectra recorded with T_2_-filter (Pulse sequence: cpmgpr1d, ns = 128) blood serum sample from HC (*black*), and BD group (*gray*). Assignments: Lactate (δ 1.33 and 4.11), glucose (δ 3.41, 3.46, 3.77 and 3.82), leucine (Leu; δ 1.71 and 3.73), proline (Pro; δ 3.34), glycerylphosphocholine (GPCho; δ 3.24), *myo*-inositol (δ 3.25), creatine (δ 3.03), glutamine (Gln; δ 2.44), glutamate (Glu; δ 2.14), lipids (δ 1.29 and 2.04), lysine (Lys; δ 1.68), alanine (Ala; δ 1.47), valine (Val; δ 1.04)

**Fig. 2:**
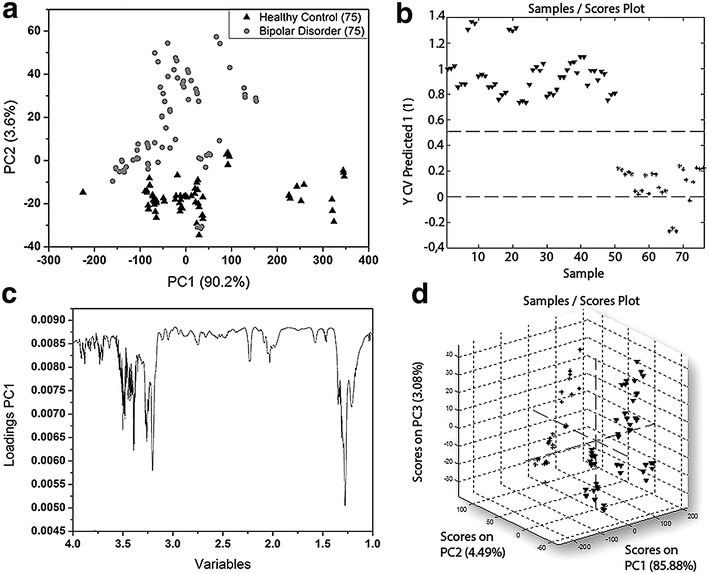
Metabolomics discriminate bipolar disorder patients from healthy individuals. **a** Plots of cross-validated PCA scores, 50 samples (25 BD + 25 HC) is equivalent to 150 spectra (75 BD + 75 HC). **b** 2D samples scores plot of PLS-DA, 76 samples (26 BD + 50 HC) is equivalent to 228 (78 BD + 150 HC). **c** Loadings Graph of spectral region **δ** 1.00 to 4.40 used in chemometrics. **d** 3D samples scores plot of PLS-DA

To explore all the potential differences in the metabolic profiles between the BD and HC groups, the ^1^H-NMR spectra (Fig. 1a) were subjected to PCA, i.e., the spectral data obtained for 76 blood serum samples that were acquired as 228 spectra were used in PCA. T_2_-edited spectra were not used in chemometrics analysis because these spectra show altered peak areas in relation to the peaks of ^1^H-NMR spectra. Based on the PCA results, it was possible to observe a distinction of the samples into two groups: BD and HC using the spectral region **δ** 1.00 to 4.40 (Fig. 2).

Considering the PCA results and the previous observations about the spectral regions not relevant for analyses, PLS-DA was performed using the same spectral range employed in the PCA. PLS-DA of ^1^H NMR spectroscopy data revealed an excellent separation between BD and HC groups.

**Fig. 3:**
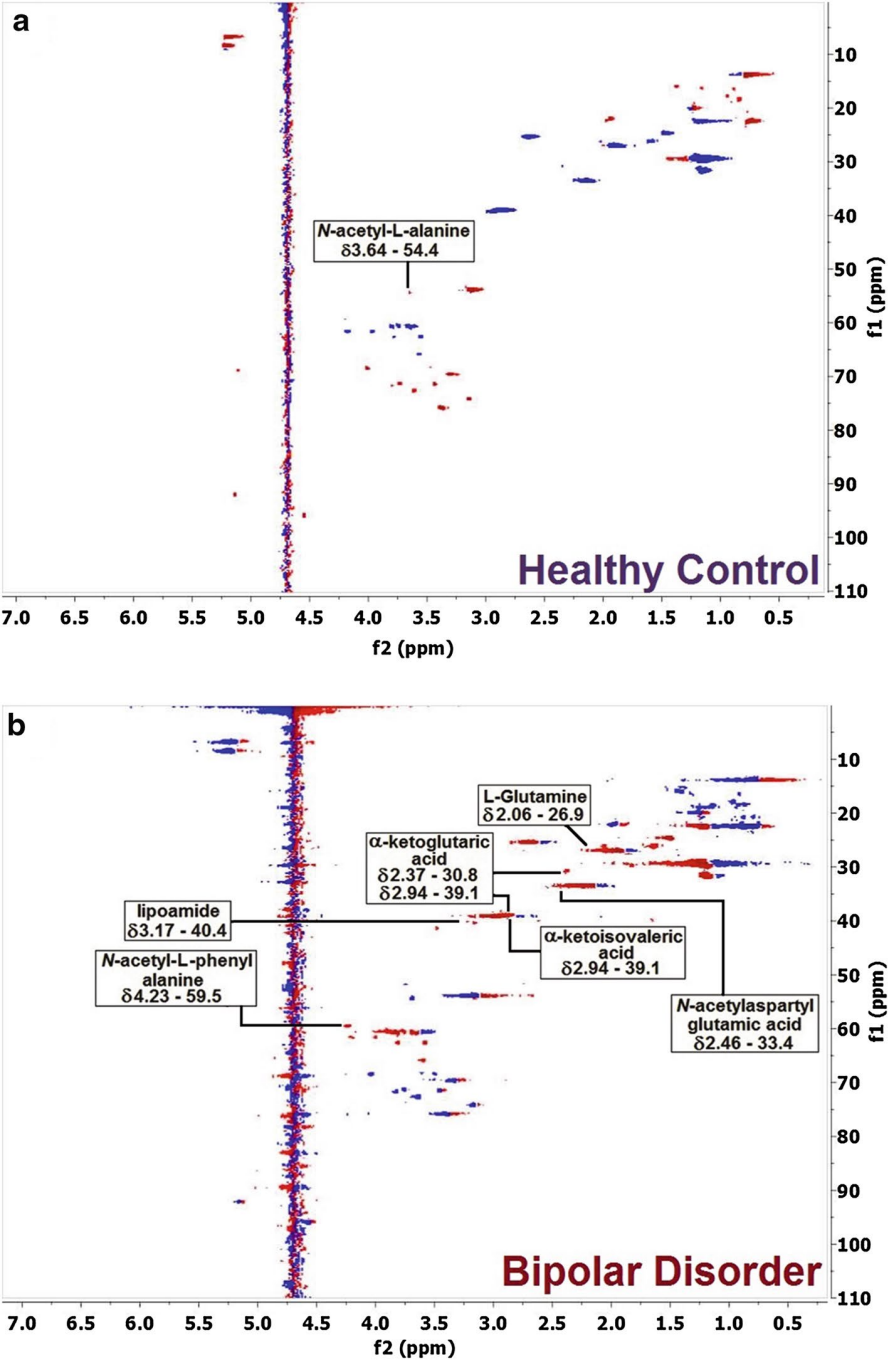
Two-dimensional NMR Spectra: Contour maps of HSQC (pulse sequence: hsqcedetgpsp.3) blood serum sample from (**a**) HC (ns = 64, SD = 16), and **b** BD group (ns = 64, SD = 16)

**Fig. 4:**
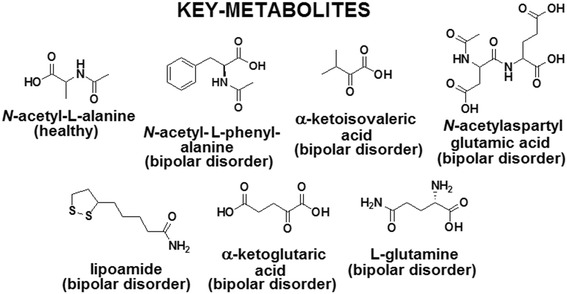
Chemical structures of seven key-metabolites (1 from HC group + 6 from BD group) identified by 2D NMR spectroscopy

Through the assignments from ^1^H-NMR edited with T_2_ filter (Fig. 1b) and the correlations from the HSQC contour maps (Fig. 3), it was possible to compare the obtained data with databases. Thereby, based mainly on spectral data (chemical shift, coupling constants, and multiplicity) and in accordance with biochemical knowledge, seven key-metabolites (Fig. 4) were found to be crucial for the two-group separation. Furthermore, it was observed that there are significant differences in some metabolite concentrations when the two investigated groups were compared, in which important significant variations in some metabolites were observed, and compounds were then identified through T_2_-edited spectra. The blood serum samples from the BD group had shown to be richer in lipids, whose amounts were up to 1/3 higher compared with those from the HC group. Also, metabolites such as d-glucose, l-alanine, and lactate were more pronounced in BD group compared with HC group. All these metabolites were found to be more abundant in BD group than in HC group, as their peaks areas were at most presenting a ratio of about 3:2 (Additional file 1: Table S3).

## Discussion

^1^H-NMR-based metabolomics were used to chemometric analysis. PCA and PLS-DA analyses were employed to examine the metabolic profiles of blood serum of individuals with BD compared with HC volunteers. The loading plot (Fig. 2) highlighted the most significant variables with the highest loading values, which corresponded to the spectral region from **δ** 1.00 to 4.40. The ^1^H-NMR spectra of BD blood serum samples are characterized by overlaps that resulted in broad and intense peaks. So, we acquired T_2_-edited ^1^H-NMR spectra (Fig. 1b) to achieve a better spectral resolution and assign peaks referred to the metabolites with the low molecular weight (<1500 Da), such as: l-valine, l-alanine, and creatine. The peaks assignments were performed in comparison with chemical shifts values that were published previously (Misra and Bajpai 2009). It is important to mention that the peaks in T_2_-edited spectra are not appropriate for integration. Through comparative analysis of the 2D NMR spectra, we observed important differences in metabolic profiles among serum samples from the two, BD and HC, groups. These differences are indicated in Fig. 3, in which it is possible to observe that some metabolites such as lipoamide, l-glutamine, among others, were detected only in BD group, while *N*-acetyl-l-alanine was observed only in HC group (Fig. 4).

The metabolites that had their peaks assigned based on the 2D NMR and that were classified as “healthy” means that these metabolites have a higher concentration in blood serum samples from the HC (healthy control) group compared with the BD group individuals (Additional file 1: Table S1). The same reasoning can be applied to the BD group. However, it is important to remark that we have not quantified the identified biomarkers up to the present moment.

Despite the large overlapping between the peaks in the ^1^H NMR spectra, it was possible to estimate the concentration ratios for some metabolites, such as d-glucose, l-alanine, lactate, and lipids, through comparison of the mean value rates of peaks integration of ^1^H-NMR spectra (Additional file 1: Table S3). For comparison of HC and BD profiles, the greatest variation in concentration of metabolites was observed for the lipids.

Partially, our results are in accordance with numerous previous studies that have demonstrated changes in brain metabolites in BD by in vivo ^1^H NMR. Davanzo et al. (2001) have shown an increase in *myo*-inositol in anterior cingulate cortex in children with BD. Acute lithium(I) treatment was associated to a significant reduction in the *myo*-inositol/creatine ratio, especially among responders to this medication (Davanzo et al. 2001). This result was also reproduced in adults (Machado-Vieira et al. 2015). Several other studies produced mixed results with choline, GABA, and glutamate, probably influenced by methodological issues, chosen brain areas or differences in subpopulations of individuals with the disorder (Bustillo 2013).

The increase of risk of hyperlipidemia in BD patients was reported by Hsu et al. (2015), which corroborates with our results, in which increases in the lipids levels in BD were also noticed (Fig. 1b). Furthermore, currently Yoshimi et al. (2016) studied the metabolism of BD patients through analysis of blood serum samples using capillary electrophoresis-time-of-flight mass spectrometry (CE-TOF/MS). They reported changes in amino acids metabolism (valine, alanine, glutamine) and metabolites belonging to the citric acid and urea cycles, such as the α-ketoglutarate and *N*-acetylglutamic acid whose levels were increased in BD patients. In our analyses, the presence of α-ketoglutaric acid, α-ketoisovaleric acid and *N*-acetyl-l-aspartyl-l-glutamic acid (NAAG) was observed, as pointed in Fig. 4.

Glutamate and glutamine are well-known markers (Manji et al. 2003). Also, Pålsson et al. (2015) reported the increase in the levels of these metabolites in blood serum and cerebrospinal fluid (CSF) of BD patients using HPLC with fluorescence detection. This increase can be due to mitochondrial dysfunction or alterations in the metabolism of the cell (Hsu et al. 2015).

However, to our knowledge, the ^1^H-NMR spectroscopy-based metabolomics analysis of serum was less studied. Sussulini et al. (2009) investigated differential metabolites in human serum sample of patients with BD (*n* = 25) under different drug treatments: lithium(I) (*n* = 15) versus other medications (*n* = 10). This strategy showed significant potential for exploring pathophysiological and toxicological features involved in BD. The investigated groups (HC and patients with BD under different treatments) could be distinguished according to their metabolic profiles, and the main differential metabolites found were glycoprotein lipids, mono- and polyunsaturated lipids, acetate, choline, glutamate, and *myo*-inositol.

The results of this research should be interpreted at light of some limitations. First, the small sample size precludes subgroup analysis including potential medication effects in metabolic profile. In addition, the inclusion of only euthymic individuals makes impossible to know which metabolic changes are related to traits and which are related to mood states in BD. The different proportion of men and women between the BD and HC groups is a result of a female predominance in the mental health service where the study was conducted. On the other hand, no significant differences in metabolic profile between female and male were noticed here. Strengths of the current study included the very careful selection of cases and controls, including the inclusion of patients without any significant comorbidities and HCs without any evidence of personal or family mental illness history.

## Conclusion

Considering the main results of this study, we can conclude that ^1^H NMR spectroscopy-based metabolomics analysis of serum could be a useful strategy to investigate the pathophysiology of BD, as well as to identify and validate potential biomarkers for diagnosis and/or for treatment follow-up.

In this study, it was possible to the identify seven key-metabolites, which showed to be in accordance with some other works described in the literature that used different analytical platforms for monitoring BD. Although the greatest differences between the two studied groups were observed in lipid ratios (more abundant in the BD group), due to the overlapping, some integration of the peak areas can not be taken as sufficiently precise for quantification of some metabolites. Nevertheless, it is important to mention that the spectral region used was similar to the work of Sussulini et al. (2009) indicating a greater accuracy in this spectral range when looking for the biomarkers and also showing the high reproducibility of the NMR spectroscopy technique in metabolomic profiling of BD individuals.

## Additional file

**Additional file 1.** HSQC and Description of 1H-NMR spectra for individuals with bipolar disorder and for healthy controls, HSQC contour map correlations and identification of possible key metabolites.
