# Supplementary material for: 1H-NMR, 1H-NMR T2-edited, and 2D-NMR in bipolar disorder metabolic profiling
Source: Int J Bipolar Disord. 2017 Jun 4;5:23. doi: 10.1186/s40345-017-0088-2 (PMC5457743; doi:10.1186/s40345-017-0088-2)
Supplement: Supplementary file 1 — Additional file 1. HSQC and Description of 1H-NMR spectra for individuals with bipolar disorder and for healthy controls, HSQC contour map correlations and identification of possible key metabolites. [file 40345_2017_88_MOESM1_ESM.docx]

**Supporting Information**

**^1^H NMR-based Metabolomics in Bipolar Disorder Profiling**

**Sumit Sethi^1^, Mariana Pedrini^1^, Lucas B. Rizzo^1^, Maiara Zeni-Graiff^1^, Caroline Dal Mas^1,2^,Ana Cláudia Cassinelli^3^, Mariane N. Noto^1,3^, Elson Asevedo^1^, Quirino Cordeiro^3^, João G. M. Pontes^4^, Antonio J. M. Brasil^4^, Acioly Lacerda^1^, Mirian A Hayashi^1,2^, Ronei Poppi^5^, Ljubica Tasic^4^, Elisa Brietzke^1,^***

**Figure S1 -** HSQC contour map of blood serum sample of control group.

**Figure S2 -**^1^H NMR spectra edited with *T_2_* filter of a control group.

**Figure S3 -** HSQC contour map of blood serum sample of patient with bipolar disorder.

**Figure S4 -**^1^H NMR spectra edited with *T_2_* filter of patient with bipolar disorder.

**Table S1 -** HSQC contour map correlations (**δ_H_**-**δ_C_**).

| **HSQC (control)** | | **HSQC (bipolar disorder)** | |
| --- | --- | --- | --- |
| **Chemical Shift (^1^H)** | **Chemical Shift (^13^C)** | **Chemical Shift (^1^H)** | **Chemical Shift (^13^C)** |
| 0.75 | 22.4 | 0.66 | 22.5 |
| 0.76 | 20.7 | 0.70 | 22.4 |
| 0.78 | 13.8 | 0.72 | 13.9 |
| 0.85 | 18.3 | 0.77 | 29.6 |
| 0.88 | 16.5 | 0.78 | 20.6 |
| 0.90 | 18.9 | 0.78 | 22.4 |
| 0.94 | 18.0 | 0.80 | 13.8 |
| 0.97 | 22.4 | 0.87 | 18.4 |
| 1.05 | 22.4 | 0.87 | 20.7 |
| 1.16 | 16.3 | 0.90 | 16.5 |
| 1.17 | 31.7 | 0.96 | 17.8 |
| 1.18 | 29.4 | 1.05 | 29.4 |
| 1.19 | 22.5 | 1.07 | 22.5 |
| 1.19 | 29.3 | 1.09 | 31.7 |
| 1.23 | 19.2 | 1.14 | 18.7 |
| 1.23 | 20.0 | 1.19 | 31.7 |
| 1.26 | 20.1 | 1.20 | 29.5 |
| 1.30 | 29.6 | 1.20 | 31.5 |
| 1.37 | 16.1 | 1.21 | 22.4 |
| 1.42 | 29.5 | 1.21 | 29.4 |
| 1.47 | 24.6 | 1.22 | 19.9 |
| 1.54 | 26.1 | 1.22 | 29.2 |
| 1.60 | 26.3 | 1.23 | 20.9 |
| 1.73 | 27.0 | 1.25 | 20.0 |
| 1.91 | 27.0 | 1.34 | 19.9 |
| 1.94 | 21.9 | 1.40 | 16.0 |
| 2.00 | 27.0 | 1.50 | 24.6 |
| 2.14 | 33.5 | 1.64 | 26.2 |
| 2.34 | 30.9 | 1.82 | 26.9 |
| 2.65 | 25.4 | 1.82 | 29.7 |
| 2.92 | 39.1 | 1.91 | 22.1 |
| 3.12 | 53.9 | 1.94 | 27.0 |
| 3.14 | 74.1 | 1.96 | 21.9 |
| 3.19 | 53.9 | 2.05 | 26.1 |
| 3.31 | 69.6 | 2.06 | 26.9 |
| 3.32 | 75.9 | 2.09 | 33.5 |
| 3.38 | 75.8 | 2.16 | 33.5 |
| 3.44 | 71.5 | 2.36 | 33.5 |
| 3.44 | 71.4 | 2.37 | 30.8 |
| 3.46 | 68.3 | 2.40 | 33.5 |
| 3.55 | 62.6 | 2.46 | 33.4 |
| 3.56 | 65.9 | 2.55 | 25.4 |
| 3.62 | 72.7 | 2.58 | 25.4 |
| 3.63 | 60.7 | 2.68 | 25.4 |
| 3.64 | 54.4 | 2.92 | 53.9 |
| 3.73 | 71.4 | 2.94 | 39.1 |
| 3.74 | 60.6 | 3.10 | 53.9 |
| 3.78 | 62.6 | 3.13 | 74.1 |
| 3.79 | 60.8 | 3.14 | 53.9 |
| 3.80 | 71.7 | 3.17 | 40.4 |
| 3.81 | 60.6 | 3.17 | 74.1 |
| 3.97 | 61.6 | 3.29 | 69.5 |
| 4.01 | 68.4 | 3.32 | 75.8 |
| 4.17 | 61.5 | 3.33 | 69.6 |
| 4.55 | 95.9 | 3.35 | 53.9 |
| 4.67 | 0.0 | 3.35 | 75.8 |
| 4.69 | 0.0 | 3.40 | 71.4 |
| 5.11 | 68.9 | 3.40 | 75.8 |
| 5.13 | 92.1 | 3.46 | 71.5 |
| 5.18 | 6.7 | 3.49 | 41.4 |
| 5.20 | 9.0 | 3.49 | 68.4 |
| 5.21 | 8.4 | 3.54 | 60.6 |
|  |  | 3.58 | 62.5 |
|  |  | 3.59 | 65.9 |
|  |  | 3.60 | 60.6 |
|  |  | 3.64 | 72.7 |
|  |  | 3.65 | 60.8 |
|  |  | 3.68 | 54.3 |
|  |  | 3.69 | 60.6 |
|  |  | 3.76 | 71.4 |
|  |  | 3.76 | 60.5 |
|  |  | 3.81 | 62.6 |
|  |  | 3.82 | 60.7 |
|  |  | 3.82 | 71.7 |
|  |  | 4.00 | 61.6 |
|  |  | 4.03 | 68.5 |
|  |  | 4.23 | 59.5 |
|  |  | 4.57 | 95.9 |
|  |  | 4.66 | 0.1 |
|  |  | 4.69 | 0.0 |
|  |  | 5.12 | 6.7 |
|  |  | 5.12 | 92.1 |
|  |  | 5.15 | 8.4 |
|  |  | 5.16 | 92.1 |
|  |  | 5.21 | 6.7 |
|  |  | 5.22 | 6.8 |
|  |  | 5.24 | 8.5 |

**Table S2-**Assignments of HSQC contour maps correlations for possible key-metabolites.

| **Metabolite** | **Theoretical correlations** | **Experimental (Healthy)** | **Experimental**  **(BD)** | **References** |
| --- | --- | --- | --- | --- |
|   Propionic acid | 2.17 - 33.3 (2)  1.04 - 12.8 (3) | 2.14- 33.5  0.78 - 13.8 | 2.16- 33.5  0.72 - 13.9 | BMRB  [bmse000179](http://www.bmrb.wisc.edu/metabolomics/mol_summary/show_data.php?molName=propionic_acid&id=bmse000179)  HMDB  [**(HMDB00237)**](http://www.hmdb.ca/metabolites/HMDB00237)  PRIMe  (C00163) |
|   selenomethionine | 3.83 - 57.4 (5)  2.21 - 33.5 (7)  2.62 - 21.8 (6)  2.02 - 5.90 (8) | 3.81- 60.6  2.14- 33.5  2.65- 25.4  --- | 3.82 - 60.7  2.16 - 33.5  2.68 - 25.4  --- | BMRB  [bmse000291](http://www.bmrb.wisc.edu/metabolomics/mol_summary/show_data.php?molName=Selenomethionine&id=bmse000291) |
|   ethanolamine | 3.80 - 60.5 (1)  3.13 - 44.1 (2) | 3.81- 60.6  --- | 3.76- 60.5  3.17-40.4 | HMDB  [**(HMDB00149)**](http://www.hmdb.ca/metabolites/HMDB00149)  PRIMe  (C00189) |
| Amygdalin | 4.06 - 71.0 (6, 7)  3.83 - 63.3 (6, 7) | 4.01 - 68.4  3.81 - 60.6 | 3.82-71.7  3.81 - 62.6 | BMRB  [bmse000139](http://www.bmrb.wisc.edu/metabolomics/mol_summary/show_data.php?molName=amygdalin&id=bmse000139) |
| Sucrose | 5.39 - 94.7 (1)  3.87 - 84.1 (2)  4.20 - 79.2 (3)  4.03 - 76.7 (4)  3.82 - 75.1 (5)  3.74 - 75.3 (6)  3.54 - 73.7 (7)  3.47 - 71.9 (8)  3.66 - 64.0 (9)  3.80 - 62.7 (10)  3.80 - 65.0 (11) | 5.13 - 92.1  ---  ---  ---  3.80 - 71.7  3.73 - 71.4  3.62 - 72.7  3.44 - 71.4  3.56 - 65.9  3.81 - 60.6  4.01 - 68.4 | 5.12 - 92.1  ---  ---  ---  3.82 - 71.7  3.76 - 71.4  3.64 - 72.7  3.46 - 71.5  3.59 - 65.9  3.82 - 60.7  4.03 - 68.5 | BMRB  [bmse000119](http://www.bmrb.wisc.edu/metabolomics/mol_summary/show_data.php?molName=sucrose&id=bmse000119)  PRIMe  (C00089) |
|   *N*-acetyl L-alanine | 4.11 - 53.6 (2)  2.00 - 24.5 (9)  1.31 - 19.9 (1) | 3.64 - 54.4  2.00 - 27.0  1.26 - 20.1 | --- | BMRB  [bmse000157](http://www.bmrb.wisc.edu/metabolomics/mol_summary/show_data.php?molName=N_acetyl_L_alanine&id=bmse000157) |
|   Glucose | 5.21 - 94.7 (1)  4.63 - 98.5 (1)  3.88 - 63.4 (6)  3.82 - 74.1 (2-5)  3.70 - 75.4 (2-5)  3.52 - 74.1 (2-5)  3.46 - 78.5 (2-5)  3.23 - 76.8 (2) | 5.13 - 92.1  ---  4.01 - 68.4  3.80 - 71.7  3.62 - 72.7  3.80 - 71.7  3.38 - 75.8  3.32 - 75.9 | --- | BMRB  [bmse000015](http://www.bmrb.wisc.edu/metabolomics/mol_summary/show_data.php?molName=D_glucose&id=bmse000015) |
| *N*-acetyl-D-phenyl alanine | 7.29 - 131.4 (9,10)  7.37 - 130.7 (12-14)  7.31 - 129.3 (12-14)  4.45 - 58.9 (5)  3.20 - 40.1 (6)  2.93 - 40.1 (6)  1.92 - 24.1 (15) | --- | 4.23 - 59.5  3.17 - 40.4  2.94 - 39.1  1.91 - 22.1 | BMRB  [bmse000479](http://www.bmrb.wisc.edu/metabolomics/mol_summary/show_data.php?molName=N_acetyl_D_Phenyl_alanine&id=bmse000479)  HMDB  [**(HMDB00512)**](http://www.hmdb.ca/metabolites/HMDB00512) |
| lipoamide | 3.40 - 57.1 (1)  2.99 - 39.0 (2)  2.93 - 39.0 (3)  2.26 - 40.8 (4)  1.15 - 35.8 (5)  1.71 - 40.8 (6)  1.58 - 26.2 (7)  1.56 - 35.5 (8)  1.39 - 29.5 (9) | --- | 3.35 - 53.9  ---  2.94 - 39.1  3.17 - 40.4  1.20 - 31.5  ---  1.64 -26.2  2.09 - 33.5  1.22 - 29.2 | BMRB  [bmse000769](http://www.bmrb.wisc.edu/metabolomics/mol_summary/show_data.php?molName=alpha_lipoamide&id=bmse000769)  HMDB  [**(HMDB00962)**](http://www.hmdb.ca/metabolites/HMDB00962)  PRIMe  (C00248) |
| *N*-Acetylaspartyl-glutamic acid | 4.74 - 51.2 (1)  4.42 - 53.1 (2)  2.82 - 36.5 (3)  2.70 - 36.5 (4)  2.40 - 30.8 (5)  2.19 - 27.7 (6)  1.99 - 22.4 (7)  1.95 - 27.7 (8) | --- | ---  4.23 - 59.5  2.94 - 39.1  2.46 - 33.4  2.37 - 30.8  2.06 - 26.9  1.91 - 22.1  1.94 - 27.0 | HMDB  [**(HMDB01067)**](http://www.hmdb.ca/metabolites/HMDB01067) |
|   α-ketoisovaleric acid | 3.03 - 39.5 (1)  1.11 - 18.8 (2) | --- | 2.94 - 39.1  1.14 - 18.7 | HMDB  [**(HMDB00019)**](http://www.hmdb.ca/metabolites/HMDB00019) |
| α-ketoglutaric acid | 2.33 - 30.9 (1)  2.89 - 36.2 (2) | --- | 2.37 - 30.8  2.94 - 39.1 | BMRB  [bmse000064](http://www.bmrb.wisc.edu/metabolomics/mol_summary/show_data.php?molName=alpha_ketoglutaric_acid&id=bmse000064) |
| L-glutamine | 2.13 - 28.9 (5)  2.44 - 33.5 (6)  3.76 - 56.8 (2)  2.45 - 28.9 (5)  3.77 - 28.9 (5)  2.13 - 33.5 (6)  2.13 - 56.8 (2) | --- | 2.06 - 26.9  2.40 - 30.5  3.68 - 54.3  2.55 - 25.4  ---  2.16 - 33.5  2.92 - 53.9 | BMRB  [bmse000038](http://www.bmrb.wisc.edu/metabolomics/mol_summary/show_data.php?molName=L_glutamine&id=bmse000038)  PRIMe  (C00064) |
